# The development and validation of a new resilience inventory based on inner strength

**DOI:** 10.1038/s41598-023-29848-7

**Published:** 2023-02-13

**Authors:** Tinakon Wongpakaran, Tong Yang, Pairada Varnado, Yupapan Siriai, Zsuzsanna Mirnics, Zsuzsanna Kövi, Nahathai Wongpakaran

**Affiliations:** 1 Department of Psychiatry, Faculty of Medicine, Chiang Mai University, 110 Intawaroros Rd. Tambon Sriphum, Amphoe Mueng, Chiang Mai, 50200 Thailand; 2 Master of Science Program (Mental Health), Graduate School, Chiang Mai University, 239 Huaykaew Rd., Tambon Suthep, Amphoe Mueng, Chiang Mai, 50200 Thailand; 3 Department of Personality and Health Psychology, Institute of Psychology, Károli Gáspár University of the Reformed Church, Bécsi street 324, Budapest, H-1037 Hungary; 4 Centre of Specialist Postgraduate Programmes in Psychology, Institute of Psychology, Károli Gáspár University of the Reformed Church, Bécsi street 324, Budapest, H-1037 Hungary

**Keywords:** Psychology, Health care

## Abstract

There are a number of resilience scales with good psychometric properties. However, the various scales differ in their item content in accordance with the model of resilience the developer had in mind. Culture is one of the reasons for the difference. Thailand, one of the Buddhist cultures, has a different view on resilience compared with Western culture. This study aimed to develop and validate a resilience inventory created based on the inner strength concept using a confirmatory factor analysis (CFA) and Rasch measurement model. The resilience inventory (RI) was developed by creating new items representing inner strengths attributed to resilience. The inner strength was adopted to form the resilience construct, including perseverance, wisdom, patience, mindfulness, loving-kindness and equanimity. In addition, face and content validity were examined by experts in both mental health and Buddhism. The final RI comprised nine items with a 5-point Likert-type scale. The RI-9 was completed by 243 medical students who participated in the study, along with other measurements, i.e., Inner Strength-Based Inventory (iSBI), measuring the ten characteristics of perfection or inner strength, and the Core Symptom Index, measuring anxiety, depression and somatization symptoms. CFA, internal consistency and the Polytomous Rasch rating model were used to investigate the RI-9 construct validity. The mean age of the participants was 22.7 years (SD, 0.8); one-half were male (50%). The RI-9 construct demonstrated item hierarchy as follows: perseverance, patience (tolerance), mindfulness and equanimity, wisdom and loving-kindness. CFA showed that the unidimensional model fitted the data well. Rasch analysis showed no misfitting items and local dependence. The reliability of the person and item was good, and no disordered threshold was observed. Two items were found to exhibit differential item functioning due to sex. RI-9 scores were significantly related to all ten strengths from the iSBI, whereas they were negatively related to depression, anxiety, somatization and interpersonal difficulties. The RI-9 demonstrated validity and reliability. It constitutes a promising tool for outcome assessment in nonclinical populations. Further investigation on external validity as well as psychometric validation in other different cultures, should be encouraged.

## Introduction

Resilience is defined as the process of adapting well in the face of adversity, trauma, tragedy, threats or stress including the capacity to “bounce back” from these challenges^1^. Despite the heterogeneity of study populations and instruments studies, a systematic literature search has revealed that higher levels of resilience are related to fewer mental health problems^2^. Evidence can be recently found with individuals who can survive psychological distress despite the severe psychological impact of the COVID-19 pandemic^3^.

No consensual theory regarding resilience has been reached. It can be viewed differently based on a variety of perspectives^2,4^. Older individuals may note that sufficient physical activity, self-sufficiency, perseverance and meaning of life may be considered the constructs of the resilience concept^5^, whereas self-esteem and perceived family support may be considered when defining resilience among children and adolescents.

In addition to age, culture also plays important role in characterizing resilience^6^. In Western culture, resilience may be related to possessing strong internal resources, having an optimistic or positive affect, maintaining events in perspective, developing high self-esteem and high self-efficacy, maintaining positive interpersonal relationships and balancing a willingness to extend oneself to others^7–10^, Character strengths such as hope and gratitude that help to confront loss were considered a part of resilience^11^.

In some mixed culture, e.g., Suriname, five strengths were found to be associated with resilience, including religiousness, hope, harmony, acceptance and perseverance^12^.

In Thailand, people consider resilience as *viriya* (perseverance), based on the inner strength or the ten perfections in Buddha’s teaching^13,14^. One striking and famous example of resilience known to Thai people is a story of Mahajanaka authored by His Majesty the Late King Bhumibol Adulyadej^15^.

Based on the ten perfection concepts, measured by the inner Strength-Based Inventory (iSBI)^16,17^, resilience is hypothesized to have the construct of a composited strength derived from the iSBI. The resilience process would start from the feeling of loving and kindness towards oneself and others, a can-do attitude or a wise attitude/belief to overcome obstacles (wisdom), a calming state using mindfulness and meditation, as well as tolerance or patience to block oneself from surrendering and being discouraged. Equanimity is then attained to maintain the emotional equilibrium ; after the state of mind is stabilized, goals and plans are established, and finally the plan is executed with continual effort (perseverance) until the goal is achieved^13,14^.

As aforementioned, a consensus around the definition of resilience remains lacking^18^ as measurements of resilience are multifold. A meta-analysis demonstrated that 21 resilience scales were developed measuring different sets of resilience domains^19^.

One common concept of resilience involves adaptation, e.g., the means to cope with stress^20^, the ability to bounce back or recover from stress^21^, the process of adaptation^22^, or different resources of adaptation, e.g., perceived social support^23^, psychological hardiness commitment, control and challenge^24^. Other concepts of resilience involve personal competence, trust/tolerance/strengthening effects of stress, acceptance of change and secure relationships, control, spiritual influences^20,25^, social competence, family coherence^26^, creativity, humor, initiative, values orientation^27^, equanimity, perseverance, self-reliance, meaningfulness, existential aloneness^28^, self-esteem, interpersonal control^29^, confident optimism, productive and autonomous activity, interpersonal warmth and skilled expressiveness^30^.

Looking at resilience using the inner strength’s perspective and related to mindfulness, patience, equanimity, wisdom, and perseverance provide us with a basis for a new inventory. As a matter of fact, some of these strengths have been accepted as a part of resilience^31–34^. A new measurement would provide us with more choices because different measurements may capture different magnitudes of association with the interested outcome. This study aimed to develop and test the psychometrics of a new resilience inventory based on inner strength or the ten perfections concept, and its psychometric properties, including construct validity, concurrent validity, convergent validity and internal consistency using CFA and Rasch model. We hypothesized that the new measurement demonstrated by both CFA and Rasch models was unidimensional, acceptably reliable, and had concurrent validity with other measurements.

## Materials and methods

### Study design

This study employed a cross-sectional survey regarding the strength of medical students, which was a part of an academic activity during psychiatry clerkships for 5th -year medical students at Chiang Mai University, Thailand. The researchers decided to use a medical student sample as a relatively more homogeneous group than diverse subjects to avoid the possibility of item bias due to sub-population. Data were collected from 2015 to 2018, totaling 243 of 365 (66.6%) participants comprising fifth-year medical students at Chiang Mai University, Thailand.

The sample comprised 50.2% females, having a mean age of 22.7 ± 0.9 years. Study approval was obtained from the Research Ethics Committee of the Faculty of Medicine, Chiang Mai University, Thailand.

### Steps in developing the instrument

In item writing and selection procedures, the details were designed as easily understood and unequivocal for lay individuals regardless of education level. First, the author (NW) gathered sixty-five items constituting resilience and generated the representative items based on related studies that were comprehensibly interpretable within Buddhist culture, including competence, self-esteem, hope and self-efficacy^2,4,6,35,36^, as well as the strengths derived from the ten perfections or inner strengths^16^. Subsequently, the principal authors (NW and TW) composed a draft version of the resilience items related to six strengths, i.e., wisdom, patience (tolerance), loving-kindness (self-loving), perseverance, mindfulness and equanimity^16^. The draft comprised 13 items, nine positively worded and four negatively worded. Then all 13 items were examined for content validity by the investigators; two psychiatrists, two psychologists, and one psychiatric occupational therapist, having more than ten years of experience working in mental health and were regular practitioners of inner strength development, e.g., mindfulness meditation or precept practice. The results showed that the first drafted resilience inventory showed individual item content index values ranging from 0.95 to 1.00, whereas the scale index was 0.99, indicating content acceptability.

Factor analysis, maximum likelihood with Oblimin with Kaiser Normalization rotation, was performed to explore the factor structure. Factor analysis results initially yielded two factors with eigenvalues of 6.09 and 1.8. Four negatively worded items formed the second dimension with a low Cronbach’s alpha value. We preferred it to constitute a unidimensional scale; thus, all four negative worded items were removed. Finally, only nine items remained for the resilience inventory (RI-9), for which items representing wisdom comprised the highest proportion (Table 1).

**Table 1:** The content of the 9-item resilience inventory and its based theories.

	Item short description	The inner strengths of the respective item
1	Believing obstacles can be overcome	Wisdom
2	Withstanding pressure	Patience (Tolerance)
3	Having pride in one’s ability to master challenges	Loving-kindness (Self-loving)
4	Believing one has abilities	Wisdom
5	Facing a problem makes one fight actively	Perseverance
6	Always being fully conscious about it	Mindfulness and equanimity
7	Being one of those who are talented	Wisdom
8	This obstacle gives rise to learning	Wisdom
9	This time of crisis provides an opportunity	Wisdom

### The final version of the resilience inventory (RI-9)

The RI-9 comprised nine items aiming for a unidimensional scale, meaning that all items contributed to the same construct. It employed a 5-point Likert scale, including values of 1 (does not describe me at all) to 5 (describes me very well). Item examples included, “I believe that I must overcome the obstacles I face”, and “I can withstand pressure”. The scores ranged from 1 to 45; higher scores indicated higher levels of resilience.

### iSBI: Inner strength-based inventory

The Inner Strength-Based Inventory measures the ten perfections inspired by the ten perfections found within Buddhist doctrine^13^. These characteristics are described as protective factors that drive psychological change and encourage adaptation in the individual, especially for meditation practitioners^14^. The ten measurements are truthfulness, perseverance, wisdom, generosity, morality, mindfulness/meditation, patience and endurance, equanimity, determination and loving- kindness. The characteristics are measured to determine when a person is low or high in the different characteristics, with multiple- choice responses along a five-point scale. Mean scores are totaled for each item. The person reliability was 0.86 by Rasch analysis^37^. In this sample, the person reliability (comparable to Cronbach’s alpha) was 0.70.

### Core Symptom Index

The Core Symptom Index (CSI) measurement is a self-rating tool measuring anxiety, depression, and somatization symptoms, including five items representing depression, four items representing anxiety and eight items representing somatization symptoms. The instructions of the CSI require respondents to answer these questions according to their feelings during the past week. The internal consistency calculated by Omega coefficients of CSI, anxiety, depression and somatization subscales were 0.92, 0.78, 0.88, and 0.83, respectively^38^. In this sample, Cronbach’s alpha was 0.84.

### Statistical analysis

Descriptive statistics were used for demographic data, presented as a percentage, mean and standard deviation. Correlation analysis was used to investigate criteria validity and convergent validity, e.g., the relationship between iSBI and RI-9 scores. For construct validity, exploratory factor analysis using principal axis factoring was used to identify the factor structure of the RI-9, then parallel analysis^39^ and the minimum average partial correlation (MAP)^40^ were used to confirm the factor structure. Data screening analysis was employed for factor analysis, and missing data were replaced using expectation maximization. Item responses showed all items had acceptable skewness and kurtosis (< ± 2)^41^. The CFA categorically tests a priori hypotheses about relations between observed variables and latent variables or factors. For parameter estimation, as data were ordinals, robust weighted least square means and variance adjusted were employed for estimators^42^. The CFA model was estimated using structural equation modelling, and model fit statistics were calculated to determine whether the hypothesized model fits these data. Regarding the fit indexes, a Comparative Fit Index (CFI) and a NonNormed Fit Index (NFI) or Tucker-Lewis Index (TLI) > 0.95 indicates a good model fit, while a standardized root mean square residual (SRMR), and a root-mean-square error of approximation (RMSEA) ≤ 0.6, indicates a good fit^43^. In addition, the χ^2^ statistic has been used to test the goodness of model fit and nonsignificant *p* values associated with the test statistics, indicating that the null hypothesis of a perfect fit cannot be rejected. Modification indices were added to the model after the initial analysis, and CFA were carried out using Mplus 7.4^44^.

Rasch analysis is used as a set of questionnaire items intended to be summed to render a total score. Rasch analysis can demonstrate whether the data fit the measurement model by illustrating unidimensionality, fit model expectations, and being free of DIF. The Polytomous Rasch rating model was used to analyze and explore other psychometric properties. The analyses were performed as follows: (1) examining the fit between the data and the model, (2) constructing the person-item map (Wright Map), (3) assessing the person and item reliability and separation coefficient, (4) exploring the dimensionality and (5) investigating category response.

### Examining the fit between the data and the model

To test the fit model, Chi-square fit statistics were calculated to determine the difference between the observed response and that expected by the model. These fit statistics are the outfit mean square and infit mean square. For clinical observations, a mean square value should range between 0.5 and 1.7^45^. High infit and outfit reflect underfit, meaning a lack of predictability of an item. Low infit and outfit reflect overfit, meaning over-predictability of an item. The Wright Map was constructed from the hierarchy of the person and item measures to examine item and person performances. It has been suggested that the difference between the mean value of the mean person measure should be within one logit^46^. Regarding reliability, person reliability (equivalent to Cronbach’s alpha) indicates how reproducible the person’s ability order is in this sample of persons for this set of items. The item reliability indicates the sample size is sufficient to demonstrate the item hierarchy. Values ≥ 0.8 for person reliability and ≥ 0.9 for item reliability are acceptable levels^47^. In exploring dimensionality, a principal component analysis of the residuals that remain after the linear Rasch measure has been extracted from the data set was used. If the eigenvalue associated with the first contrast < 3, the scale is considered essentially unidimensional^48^, and the standardized residual correlation is less than 0.25 to indicate local independence^49^. Category functioning was examined by analyzing category frequencies, mean measures, thresholds, and category fit statistics^50^. Thresholds were expected to increase by at least 1.4 logits but not more than five logits to show distinction among categories. Ordered categories were expected. Lastly, the differential item functioning (DIF) due to sex was tested. Both statistical tests and DIF contrast were used, and a DIF contrast < 0.64 indicated no significant concern regarding DIF^51^. All Rasch analysis was performed using the Winsteps measurement software^28^ (Winsteps Rasch Measurement, Version 5.2.5.0, Chicago, IL, USA). All other analyses, including parallel analysis, were performed using IBM SPSS, Version 22.

### Ethics approval and consent to participate

 The study was conducted according to the guidelines of the Declaration of Helsinki and approved by the Institutional Review Board (or Ethics Committee) of the Faculty of Medicine, Chiang Mai University (study code, 441/2560 and date of approval, 18 June 2017) and followed the tenets of the Declaration of Helsinki.

### Informed consent

 All patients provided informed consent to the study.

## Results

Of 243 participants, the average age was 22.7 years (SD, 0.8); one- half were male (50%). Details regarding the mean scores of the items from three measurements are shown in Table 2. In the parallel analysis, the first three eigenvalues from the raw data of the RI-9 were 4.18, 0.76, and 0.56. The corresponding first three 95th-percentile random-data eigenvalues were 1.33, 1.21, and 1.12, indicating that one factor was the best option for the structure of the RI-9. Moreover, the MAP test showed that the number of components according to the original and the revised MAP test was based on the smallest average square partial correlation of 0.0309 and the smallest average, the fourth power partial correlation of 0.0017, confirming a one—factor structure for the RI-9.

**Table 2:** Sociodemographic of the participants.

Variable	n (%) or Mean ± SD
Sex, male, n (%)	122 (50.2)
Age, Mean ± SD	22.69 ± 0.8
Resilience inventory, Mean ± SD(Min–Max)	
Believe to overcome the obstacles	4.23 ± 0.8 (1–5)
Withstand the pressure	3.82 ± 0.9 (1–5)
Pride in one’s ability to master challenges	4.44 ± 0.8 (2–5)
Believe one has an ability	4.14 ± 0.8 (1–5)
Faced with a problem made one active to fight	3.64 ± 1.0 (1–5)
Always be fully conscious about it	3.87 ± 0.9 (2–5)
Be one of those who are talented	4.02 ± 0.9 (1–5)
This obstacle gives rise to learning	4.21 ± 0.9 (1–5)
This time of crisis provides an opportunity	3.98 ± 1.0 (1–5)
Total score	36.39 ± 5.9 (16–45)
Inner-strength based inventory, Mean ± SD(Min–Max)	
Truthfulness	3.54 ± 1.0 (1–5)
Perseverance	2.92 ± 0.9 (1–5)
Wisdom	3.76 ± 0.8 (1–5)
Generosity	3.93 ± 0.7 (1–5)
Morality (5 precept)	3.26 ± 1.1 (1–5)
Mindfulness	2.09 ± 1.0 (2–5)
Patience and endurance	3.56 ± 0.9 (1–5)
Equanimity	3.81 ± 0.8 (1–5)
Determination	3.67 ± 0.8 (1–5)
Loving-kindness	3.86 ± 1.0 (1–5)
Total score	34.42 ± 4.6 (24–50)
Core Symptom Index	
Anxiety	2.27 ± 2.4 (0–7)
Depression	3.32 ± 4.0 (0–19)
Somatization	1.04 ± 2.7 (0–13)
Total mental health symptoms	6.64 ± 6.9 (0–34)

SD = Standard deviation.

Table 3 shows the factor loading coefficients ranging from 0.691 to 0.852. All estimated coefficients were significant (*p* < 0.0001). The highest factor loading was item 4 “Believing one has abilities”, whereas the lowest factor loading was item 6 “Always being fully conscious about it”.

**Table 3:** Confirmatory factor analysis results for the RI-9.

	Item short description	Est	S.E	Est/S.E	*P* value
1	Believing obstacles can be overcome	0.800	0.032	24.757	< 0.0001
2	Withstanding pressure	0.711	0.036	19.549	< 0.0001
3	Having pride in one’s ability to master challenges	0.696	0.042	16.654	< 0.0001
4	Believing one has abilities	0.852	0.025	34.187	< 0.0001
5	Facing a problem makes one fight actively	0.820	0.026	32.131	< 0.0001
6	Always being fully conscious about it	0.691	0.039	17.762	< 0.0001
7	Being one of those who are talented	0.837	0.027	31.111	< 0.0001
8	This obstacle gives rise to learning	0.782	0.034	22.771	< 0.0001
9	This time of crisis provides an opportunity	0.766	0.033	22.965	< 0.0001

Est = Estimated coefficients, SE = standard error, Fit statistics showed Chi-square = 39.717, *df* = 23, *p* = 0.017, CFI = 0.995, TLI = 0.992, RMSEA 0.056 (90% CI 0.024–0.085), SRMR = 0.023.

### Convergent validity

RI-9 scores were significantly related to all ten inner strengths. The correlation coefficients ranged from 0.152 (*p* < 0.05) to 0.439 (*p* < 0.01); the highest value was with determination, whereas the lowest value was with Loving-kindness (Table 4).

**Table 4:** Spearman rank correlation between RI-9 and each inner strength.

	RI-9	Truth	Per	Wis	Gen	Mo	Med	Pa	Eq	Det	Lov
RI-9	–	.296**	.400**	.355**	.244**	.295**	.373**	.166*	.258**	.439**	.152*
Truth		–	.199**	.103	.210**	.204**	.220**	.140*	.053	.215**	.112
Per			–	.267**	.129*	.221**	.297**	.116	.075	.473**	.086
Wis				–	.153*	.102	.176**	.184**	.022	.375**	.152*
Gen					–	.179**	.187**	.209**	.024	.208**	.289**
Mo						–	.332**	.192**	-.024	.150*	.099
Med							–	.131*	.132*	.311**	.165*
Pa								–	.149*	.138*	.229**
Eq									–	.066	.006
Det										–	.110
Lov											–

Truth = Truthfulness, Per = Perseverance, Wis = Wisdom, Gen = Generosity, Mo = Morality, Med = Meditation, Pa = patience (tolerance), Eq = Equation, Det = Determination, Lov = Loving-Kindness.

### Concurrent validity

As expected, RI-9 scores were negatively related to the scores of anxiety, somatization and depression (Table 5).

**Table 5:** Correlation between RI-9 and other variables.

	RI-9	Anxiety	Somatization	Depression	Mental health symptoms
RI-9	1	− .217**	− .378**	− .458**	− .443**
anxiety		1	.481**	.461**	.760**
somatization			1	.512**	.753**
depression				1	.889**

*p < .05, **p < .01.

Table 6 shows the item fit statistics. The logit ranged from − 1.41 to 1.25. All items were shown to have fit statistics in the required range, 0.73–1.30, indicating that overall, the nine-item scale formed a valid measure. The acceptable fit statistics indicated that the unidimensional model fitted the data well.

**Table 6:** Measure and fit statistics.

	Item short description	Measure (logit)	S.E	Infit	Outfit
1	Believing obstacles can be overcome	− 0.63	0.12	0.89	0.88
2	Withstanding pressure	0.73	0.11	1.10	1.09
3	Having pride in one’s ability to master challenges	− 1.41	0.13	1.20	1.25
4	Believing one has abilities	− 0.29	0.12	0.73	0.72
5	Facing a problem makes one fight actively	1.25	0.11	0.95	0.96
6	Always being fully conscious about it	0.57	0.11	1.15	1.15
7	Being one of those who are talented	0.10	0.12	0.88	0.84
8	This obstacle gives rise to learning	− 0.53	0.12	0.87	0.94
9	This time of crisis provides an opportunity	0.22	0.12	1.2	1.14

S.E. = Standard error, infit = information-weighted fit statistics; outfit = outlier-sensitive fit statistics.

**Figure 1:**
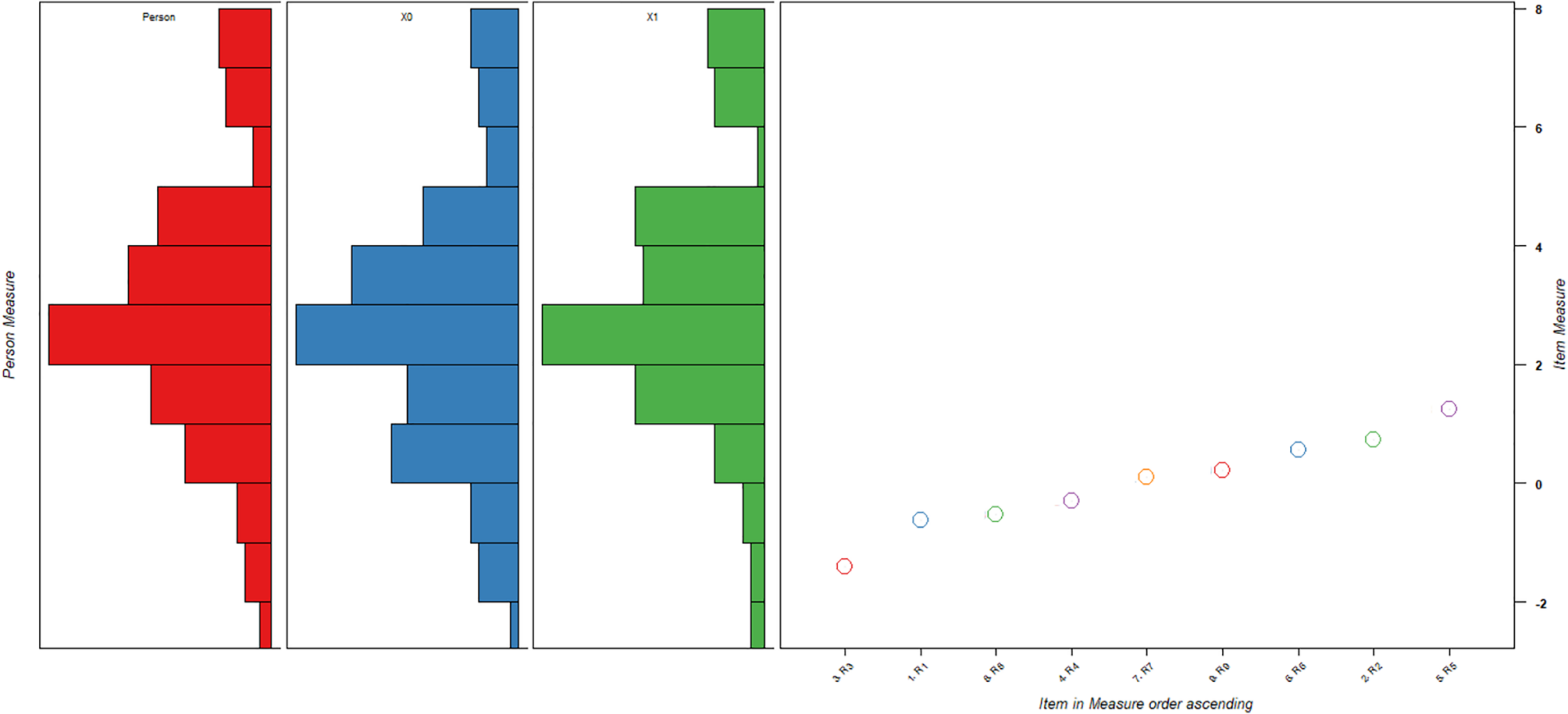
Person-item Wright Map of RI-9. Legend: Histogram representing person distribution, more able persons with high level of resilience are located at the top of the map. More difficult (strong resilience) items are located at the top of the map, X1 = Male group, X2 = Female group, Circles are item threshold, item 3 is the easiest item; item 5 is the hardest. The mean of the items is much lower than the person’s ability, indicating that RI-9 is relatively easy for this sample.

Figure 1 shows the Wright Map of the RI-9. The hardest item is item 5 (Facing a problem makes one fight active); the easiest item is item 3 (Having pride in one’s ability to master challenges). The sample and items are not well-targeted, and the map indicates that the RI-9 is relatively too easy for the sample.

### Dimensionality

The raw variance of the RI-9 explained by Rasch measures was 41.7% (expected by model 41.7%). The unexplained variance in the first contrast was 8.2% (1.76 eigenvalue units), indicating that no second dimension was observed. The disattenuated correlation between person measure was 0.896 confirming the unidimensionality.

### Local dependence

No standardized residual correlations exceeded 0.25, according to Yen^49^, indicating local independence.

### Category function

**Figure 2:**
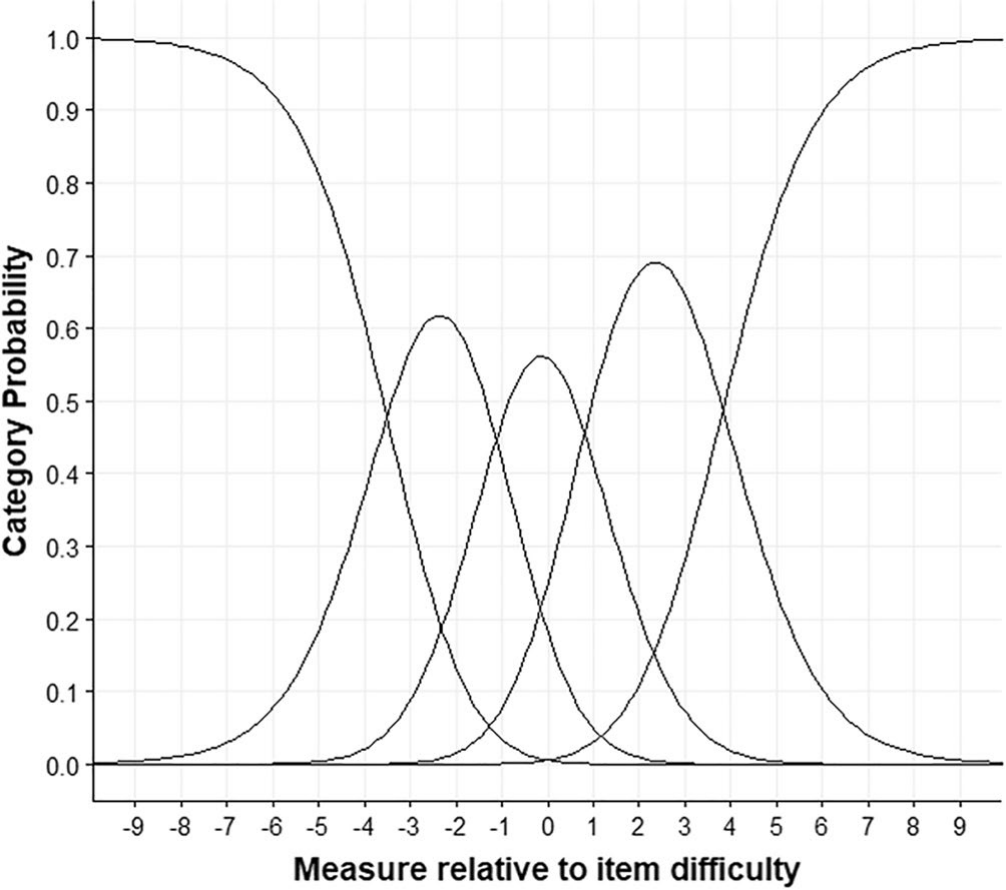
Categorical probability curves for RI-9.

Figure 2 presents the functioning of the five categories of the RI-9. All categories are well represented except for the fifth category, which presented the lowest frequency of 15 observations. The observed average measures advanced monotonically in a smooth distribution from − 2.13 to 4.30. No disordered Rasch-Andrich thresholds were observed, and none of the categories show a misfit.

### Reliability and separation statistics

The reliability analysis of the RI-9 was 0.86. Cronbach alpha person raw score “test” reliability = 0.91. Person separation was 2.44, indicating that two ranges could be confidentially differentiated. Item reliability was 0.97, and item separation was 6.04, indicating that the RI-9 has a sufficient sample of persons.

### Differential item functioning

DIF due to sex was observed in items 2 and 3. The DIF contrast values were 0.80 (Rasch-Welch *t* = 3.49, df 212, *p* < 0.001, Mental Chi-square = 17.74, *p* < 0.001) and − 0.76 (Rasch-Welch *t* =  − 2.82, df 215, *p* < 0.01, Mental Chi-square = 5.04, *p* < 0.05) for items 2 and 3, respectively. Item 2 was biased among females, whereas item 3 was biased among males.

## Discussion

This study aimed to develop and validate the resilience inventory created based on the inner-strength concept. The overall results endorsed the validity and reliability of the RI-9.

Regarding construct validity, RI-9 was proven to be a unidimensional scale from both CFA and Rasch analyses; all items represented the same construct. Furthermore, the RI-9 in this sample fitted well with Rasch measurement theory, determined by fitting and local independence items, so that respondents can be evaluated in a single overall score of resilience.

The item and person reliability were good. They are also evidence of construct validity—only two items suspecting differential item functioning. As the sample was insufficiently large to declare the DIF confidently; therefore, they were kept in the scale. The Wright Map indicated that the RI-9 items were not well-targeted but relatively easy to understand among medical students, suggesting more difficult items were required for this sample. Interestingly, the item difficulty (hierarchy) is consistent with what we hypothesized. The easiest item was related to loving-kindness, followed by wisdom, mindfulness and equanimity, patience (tolerance), and the hardest one, perseverance. The findings were consistent with the prior study regarding item difficulty hierarchy^16^.

That the RI-9 demonstrates its unidimensional construct was consistent with other unidimensional measurements such as the Brief Resilience Scale, RS-14^52,53^. In contrast, a widely used Connor-Davidson Resilience Scale (CD-RISC) showed various dimensions of classic test theory, ranging from one to three^20,54,55^. This may have contributed to the fact that CD-RISC had more items and the concept derived for the CDRS differed from the RI-9. In testing CD-RISC using Rasch analysis, however, CD-RISC showed to form an essentially unidimensional construct after two items had been removed^56^.

In addition to the unidimensional construct, RI-9 demonstrated construct validity as the item fit statistics and standardized residual correlation denoting local independence fell within an acceptable range. Items 2 and 3 appeared to have DIF due to sex. However, many subjects are required for confirmation^51^. The RI-9 was correlated with all ten strengths, not only the five hypothesized strengths but negatively correlated with negative mental health outcomes, also indicating convergent validity.

The magnitudes of correlation coefficients between iSBI subscales and RI-9, exhibited the highest value in perseverance, wisdom, truthfulness, and morality. However, other strengths such as determination are also related to the RI-9 suggesting that it should also be considered a part of the resilience construct.

The RI-9 construct contextualized six characters or inner strengths was supported by Kovi et al.’s, based the resilient score on the five-factor model using the Zuckerman-Kuhlman-Aluja Personality questionnaire. Like the present study, the resilient score was significantly related to all the ten inner strengths in Kovi’s study^17^.

Other related studies have supported perseverance as a part of resilience^12,33,34^. Another virtue that many investigators believe to be related to resilience is grit. Some use grit and resilience interchangeably. However, grit and resilience indicate an ability to handle negative feedback, setbacks and other obstacles. Grit is more like perseverance and passion toward long- term goals, whereas resilience might be viewed as an inherent attribute of grit^57^. The relationship among this triad, resilience, grit and perseverance, is, however, evident in the large body of literature^58–60^. Like resilience, grit is believed to have more attributes related to constructs such as self-efficacy, conscientiousness, perseverance, mindset, self-control, mindfulness^31,32^ and equanimity^32–34^. This notion is in good agreement with our theoretical model of resilience that should constitute resilience in addition to perseverance, that is, wisdom, patience (or tolerance), loving-kindness, perseverance, mindfulness and equanimity. However, this proposed construct of resilience inventory needs further investigation, especially in clinical samples, to verify its theoretical construct.

Other studies have reported an association between resilience and religiosity or spirituality^55,61^. Some studies reported that resilience^62^ was associated with forgiveness^52^, and lack of faith^52,63–65^. The present findings lend support to those religious contexts but shed more light on the specific religious matters, namely, the inner strengths, to see what might constitute the construct of resilience based on the ten perfections in the Buddhist concept.

It has been widely accepted that resilience is associated with or predicts mental health outcomes. Analogous to other resilience scales, the RI-9 showed significant correlations, negatively correlated with adverse mental health outcomes such as depression, stress and anxiety^52,53,55^. The present findings also endorsed a study reporting somatic symptoms during COVID-19 and found that resilience was a key predictor of somatization^66^. Finally, in line with other studies, the present study strongly supported the robust relationship between depression and resilience, as shown by the highest effect size^67,68^.

### Clinical implications

The RI-9 may be used in a variety of settings, ranging from nonclinical, e.g., students, to clinical participants (people with mental health problems), as it captures various types of inner strengths covering essential mental strengths for general people.

Moreover, in disease specific populations, e.g., physical disability, cancer, where specific resilience scales have been developed for such populations. Compared with the resilience scale that was developed for specific populations such as cancer (RS-SC)^69^, both measurements share similar components, albeit labelling it differently due to the different theoretical underpinnings. Thus, RI-9 may be applied to this population as well.

### Suggested future study

As a new measurement, RI-9 should be further tested for other validities in other cultures and in a longitudinal fashion, e.g., responsiveness, as conducted in other measurements^70–72^. Furthermore, it would be interesting to test this new resilience inventory, derived from another theoretical framework, to see whether it constitutes a trait or state construct as did the previous resilience measurement^73^.

### Strength and limitation

To the best of our knowledge, this new resilience inventory constitutes a tool capable of assessing resilience based on values from Buddhist culture. However, it should be tested in other cultures, especially non-Buddhist. Some limitations to be addressed are that the participants comprised medical students and from only one university, implying a highly selective sample that may have biased our findings. In addition, as suggested by the Wright Map, future studies need to replicate the present results, especially among clinical samples, e.g., people with mental health problems. Finally, the external validity of the RI-9 is limited as it was not tested against well-known resilience measurements, especially in Western culture. Discriminant validity was not distinctively examined. A study on this missing part should be warranted in further research.

## Conclusion

The RI-9 demonstrated its construct validity, convergent validity, and reliability through confirmatory factor analysis and the Rasch model. Resilience is constituted not only by perseverance but also by a lower construct of patience (tolerance), mindfulness and equanimity, wisdom and loving-kindness. The RI-9 demonstrated a promising tool to evaluate resilience in nonclinical populations. Further studies should extend to clinical samples as well as different cultures.

## Data Availability

The datasets used and/or analyzed during the current study available from the corresponding author on reasonable request.

## Abbreviations

RI-9: 9-Item resilience inventory
iSBI: The inner strength-based inventory
CSI: Core Symptom Index
CFA: Confirmatory factor analysis
S.E.: Standard error
Infit: Inlier-sensitive or information-weighted fit
Outfit: Outlier-sensitive fit
PCA: Principal component analysis
