# Author Correction: The development and validation of a new resilience inventory based on inner strength

**DOI:** 10.1038/s41598-023-31833-z

**Published:** 2023-04-04

**Authors:** Tinakon Wongpakaran, Tong Yang, Pairada Varnado, Yupapan Siriai, Zsuzsanna Mirnics, Zsuzsanna Kövi, Nahathai Wongpakaran

**Affiliations:** 1 Department of Psychiatry, Faculty of Medicine, Chiang Mai University, 110 Intawaroros Rd. Tambon Sriphum, Amphoe Mueng, Chiang Mai, 50200 Thailand; 2 Master of Science Program (Mental Health), Graduate School, Chiang Mai University, 239 Huaykaew Rd., Tambon Suthep, Amphoe Mueng, Chiang Mai, 50200 Thailand; 3 Department of Personality and Health Psychology, Institute of Psychology, Károli Gáspár University of the Reformed Church, Bécsi Street 324, Budapest, 1037 Hungary; 4 Centre of Specialist Postgraduate Programmes in Psychology, Institute of Psychology, Károli Gáspár University of the Reformed Church, Bécsi Street 324, Budapest, H-1037 Hungary

*Scientific Reports* 10.1038/s41598-023-29848-7, published online 13 February 2023

In the original version of this Article Zsuzsanna Kövi was incorrectly affiliated with ‘Department of Personality and Health Psychology, Institute of Psychology, Károli Gáspár University of the Reformed Church, Bécsi Street 324, Budapest 1037, Hungary.’

The correct affiliation is listed below:

Centre of Specialist Postgraduate Programmes in Psychology, Institute of Psychology, Károli Gáspár University of the Reformed Church, Bécsi street 324, Budapest, H-1037, Hungary.

The original Article has been corrected.

